# Transcriptionally Active Regions Are the Preferred Targets for Chromosomal HPV Integration in Cervical Carcinogenesis

**DOI:** 10.1371/journal.pone.0119566

**Published:** 2015-03-20

**Authors:** Irene Kraus Christiansen, Geir Kjetil Sandve, Martina Schmitz, Matthias Dürst, Eivind Hovig

**Affiliations:** 1 Department of Microbiology and Infection Control, Akershus University Hospital, Lørenskog, Norway; 2 Department of Informatics, University of Oslo, Oslo, Norway; 3 Department of Gynaecology, Jena University Hospital, Jena, Germany; 4 Department of Tumor Biology, Institute for Cancer Research, Oslo University Hospital, Oslo, Norway; 5 Institute for Cancer Genetics and Informatics, Oslo University Hospital, Oslo, Norway; Istituto Nazionale Tumori, ITALY

## Abstract

Integration of human papillomavirus (HPV) into the host genome is regarded as a determining event in cervical carcinogenesis. However, the exact mechanism for integration, and the role of integration in stimulating cancer progression, is not fully characterized. Although integration sites are reported to appear randomly distributed over all chromosomes, fragile sites, translocation break points and transcriptionally active regions have all been suggested as being preferred sites for integration. In addition, more recent studies have reported integration events occurring within or surrounding essential cancer-related genes, raising the question whether these may reflect key events in the molecular genesis of HPV induced carcinomas. In a search for possible common denominators of the integration sites, we utilized the chromosomal coordinates of 121 viral-cellular fusion transcripts, and examined for statistical overrepresentation of integration sites with various features of ENCODE chromatin information data, using the Genomic HyperBrowser. We find that integration sites coincide with DNA that is transcriptionally active in mucosal epithelium, as judged by the relationship of integration sites to DNase hypersensitivity and H3K4me3 methylation data. Finding an association between integration and transcription is highly informative with regard to the spatio-temporal characteristics of the integration process. These results suggest that integration is an early event in carcinogenesis, more than a late product of chromosomal instability. If the viral integrations were more likely to occur in destabilized regions of the DNA, a completely random distribution of the integration sites would be expected. As a by-product of integration in actively transcribing DNA, a tendency of integration in or close to genes is likely to be observed. This increases the possibility of viral signals to modulate the expression of these genes, potentially contributing to the progression towards cancer.

## Introduction

Persistent infection with human papillomavirus (HPV) has been identified as a necessary cause of cervical cancer. Among the mucosotropic HPV types, 12 are classified as carcinogenic [1], although differences exist between types in terms of carcinogenicity. HPV 16 is the most prevalent type, and together with HPV 18 these are responsible for 70–80% of all cervical cancers, followed by HPV 45, 33 and 31 [2].

The oncogenic potential of the virus is first of all associated with the expression of the viral proteins E6 and E7, which are required for immortalization and maintenance of a fully transformed phenotype [3,4]. In addition, integration of HPV into the host genome is considered as a critical genetic alteration, and is regarded as a determining event in cervical carcinogenesis [5]. Integration may result in the up-regulation of E6/E7 expression, and in that way contributes to carcinogenesis [4]. However, high levels of E6/E7 expression are not always seen [6]. Additional possible consequences of viral integration are under investigation, and the role of HPV integration in the process of tumor progression is an area of high importance in understanding the etiology of the disease.

HPV integration occurs into different genomic locations, and all loci so far reported appear to be unique and to be distributed across the whole genome. Insertional mutagenesis, in analogy to retrovirus-induced carcinogenesis, would therefore not be expected to play a significant role in cervical carcinogenesis. On the other hand, certain regions have independently been reported for integration more often than others, and there is increasing evidence for a non-random distribution of integration sites [7,8]. Clusters of integration sites have been reported in specific cytogenetic bands, for example 3q28, 8q24.21 and 13q22.1, with these currently being referred to as integration hotspots. However, these observations remain an uncharacterized phenomenon. It has been suggested that such hotspots may reflect fragile sites that are genetically unstable, or regions harboring specific cancer-related genes that may have been altered as a consequence of the viral integration. The logic of the latter is that integration is non-discriminatory and random and that those few events that by chance interfere with regions of regulatory importance more frequently cause cancer and are hence studied.

A number of HPV integration sites have been revealed within or surrounding important cancer-related genes, and it was early suggested that in some instances, HPV DNA integration may disrupt the coding region or cause deregulation of these genes [9]. In 1998, the first case of an actual gene deregulation was shown, reporting the functional inactivation of APM-1, a putative tumor suppressor gene, which had resulted from insertional mutagenesis in combination with the deletion of the second allele of the gene [10]. During recent years, more effort has been put into characterizing integration sites, and the hypothesis that the integrated virus alters the expression of adjacent genes is strengthened [11,12]. Ojesina and colleagues essentially confirmed this hypothesis by showing, for several samples, that the level of gene expression at individual HPV integration sites was significantly higher than in other tumors [13]. The increased expression of adjacent genes may be driven by copy-number gains, or by the actual integration of viral promoters, as is shown in the same study. In addition, HPV integration can disrupt adjacent genes by inducing gene rearrangements [14]. Also, in a recent study on transcriptome sequencing data from The Cancer Genome Atlas Research Network, an association between integration and expression of affected genes was reported [15].

Cancer progression is a complex and multistep process, and although the exact dynamics and consequences of HPV integration remains largely unexplored, all aspects described above are likely to be involved. It is therefore important to differentiate between the general dynamics of integration and the selective advantage bestowed upon cells in which a particular integration has triggered carcinogenesis (cells undergoing added clonal selection pressure). A general consideration is that the integration event partially is a consequence of chromosomal instability and that fragile sites, translocation break points and transcriptionally active regions are preferred sites for integration [16–19], which is likely to reflect a greater accessibility of these regions to viral integration. Additionally, selection mechanisms may act on the integrated viral sequences to drive carcinogenesis, and the overrepresentation of for example fragile sites may also indicate a greater susceptibility to integration-induced chromosomal alterations [16]. Also, repetitive elements, as interspersed nuclear elements, have been reported to be preferential sites for integration [20]. In order to further investigate these assumptions, and in a search for possible common denominators of the integration sites, 121 documented sites were subjected to rigorous statistical analyses.

## Materials and Methods

### HPV integration data

The following 121 HPV integration sites (at 119 unique genomic coordinates) were assembled and included in the analysis: Chromosomal coordinates of APOT-products (viral-cellular fusion transcripts) reported in Kraus et al., 2008 [7] (n = 74; HPV 16 (n = 15), 18 (n = 27), 31 (n = 2), 33 (n = 7), 45 (n = 23)) as well as those reported in Schmitz et al., 2012 [8] (n = 47; HPV 16 (n = 34) and 18 (n = 13)). For sites reported in Kraus et al., sequence searches were repeated using NCBI (National Centre for Biotechnology Information) Build 37 and the UCSC (University of California, Santa Cruz) hg19 (Feb 2009) human genome assemblies. The integration coordinates of the sites reported in Schmitz et al. refer to the same genome assembly. All samples investigated in the previous studies were either high-grade cervical intraepithelial neoplasia or cancer tissue samples.

As the sites included in our analysis are identified by methodology detecting active integration sites, a dataset reported in a study by Schwarz and colleagues [12], comprising both active and inactive sites, was used for result confirmation.

### Ethics statement

Tissue samples are approved for use in HPV research by the Norwegian regional committee for medical and health research ethics (REC South East). Samples from Germany were collected within the framework of a prospective study concerning the sentinel lymph node concept in cervical cancer which was approved by the institutional review committee (reference number 0175-02/00) of the Jena University Hospital. All patients provided written informed consent to use their biopsy material for further molecular analyses.

### Statistical procedures and software tools

All statistical analyses of HPV integration sites in relation to other genomic features were performed using the Genomic HyperBrowser [21,22], with all analytical steps. The relationship between HPV integration sites and genes was investigated by asking whether HPV integration sites fell more inside Ensembl gene regions than expected by chance. HPV integration sites were treated as points, i.e. occurring at a single base pair, and Ensembl gene regions were treated as segments [23] along the genome sequence. P-values were computed based on a Monte Carlo scheme, evaluating in what proportion of random Monte Carlo samples the test statistic was more extreme than the corresponding value of the test statistic for the observed data. The test statistic used was the number of HPV sites (points) falling within regions covered by genes (segments). In the null hypothesis, Ensembl genes were preserved as they were, while locations of HPV sites were randomized according to a null model where the empirical distribution of inter-point distances between HPV sites was preserved (to preserve the clustering tendency of HPV sites in the hypothesis test).

For viral integration to occur, double-strand breaks of the chromosomal DNA are necessary [4,14]. The propensity of DNA breakage varies considerably along the genome, with fragile sites being among the regions most highly exposed. Fragile regions of the human genome are, however, not completely characterized, and hence an incomplete manually curated data set, covering 55 megabases (mb) of the genome (hg19), was chosen for analysis. The relation between HPV and fragile regions was investigated by asking whether HPV integration sites occurred significantly often inside fragile regions, using the same procedure as for the HPV-gene relation.

Histones are proteins necessary for the packaging of genomic DNA and modifications (including acetylation, methylation and phosphorylation) on specific amino acid residues of the histones are involved in the regulation of gene expression [24,25]. In particular, methylation and acetylation on specific lysine residues are essential, and depending on the modification, the lysine residue, and the level of modification, transcription is either activated or repressed. Specifically, trimethylation of histone H3 on lysine 4 (H3K4me3) is associated with activation of transcription, and H3K4me3 is commonly used as a mark of active promoters. It has also been shown that genome-wide profiles of H3K4me3 can be used to suggest relevant cell types for complex trait variants [26]. In order to assess the co-occurrence of HPV integration sites and H3K4me3 histone modifications in a given cell type versus the general tendency of overlap between HPV integration sites and H3K4me3 modifications across all cell types, we applied a three-track approach: A general H3K4me3 track (track 1) was created by aggregating all H3K4me3 sites across all cell types/tissues. Enrichment (factor of co-occurrence beyond expectance) for a particular cell type was calculated as the ratio of cell-specific H3K4me3 (track 2) coverage to aggregated H3K4me3 coverage in the proximity of HPV integration sites (integration location including 10kb flanks) (track 3), divided by the corresponding ratio for the full genome. This is equivalent to evaluating whether cell-specific H3K4me3 modifications overlap with regions around HPV integration sites, more than the general tendency of H3K4me3 to do this across all cell types. The aggregated H3K4me3 track contains overlapping regions (sites from different cell types that have overlapping genome coordinates), and in such cases the coverage for a given base pair was calculated as the count of regions covering the base pair.

Statistical hypothesis testing was performed using a permutation-based approach to compute p-values. We defined a null model for which the location of individual insertion sites were uniformly randomized within their respective chromosome arms, whereas H3K4me3 sites were kept fixed. The relative enrichment, as described above, was used as a test statistic in the hypothesis testing, meaning that the test captures the tendency of HPV integration sites to co-occur with H3K4me3 of a particular cell type beyond the general (aggregate) co-occurrence of HPV integration sites and H3K4me3 across all cell types. This test statistic was calculated for the HPV sites, as well as for several Monte Carlo samples from the null model. The p-value was calculated as the proportion of Monte Carlo samples being equal to, or more extreme than, the observed test statistic. Directly testing the co-occurrence of HPV integration sites with H3K4me3 of a particular cell type (without normalizing against the aggregate H3K4me3 signal) would have resulted in substantially lower (more significant) p-values (data shown at supplementary Galaxy Page).

DNase hypersensitive sites (DHSs) are regions in the chromatin being sensitive to cleavage by the enzyme DNase, due to loss of the condensed structure of the chromatin. An open state of the chromatin is necessary for the binding of proteins such as transcription factors and hence such regions are functionally related to transcriptional activity [27]. Data for DNase hypersensitivity are available for a number of cell types [28]. These data were used to compute the enrichment of HPV integration sites within DHSs of the respective cell types, using a similar procedure as described for H3K4me3 above.

## Results

Several non-random aspects of HPV integration were discovered, both when the genomic locations were analyzed separately and when analyzed in relation to other genomic elements. Of the 119 unique integration sites, 78 (66%) occurred inside Ensembl genes, which is a significant enrichment (p = 0.004). However, considering that 54% of the genome (hg19) is covered by Ensembl gene regions (exons and introns), the enrichment is not very strong. In fact, if Refseq is used as the source of gene annotations, no preference to occur inside gene regions is seen (51 integrations inside gene regions, p = 0.4). As the differences between the Ensembl and Refseq gene collections mainly reflect differing definitions and criteria for designating a genomic region to be a gene, these results indicate no strong and robust preference for HPV to generally occur inside gene regions.

HPV sites were further found to occur preferentially inside fragile regions. Out of 119 unique integration sites, 11 occurred inside a fragile region, which is significantly more than expected (p = 0.002).

### Co-occurrence of HPV integration sites and H3K4me3 histone modifications in a given cell type

**Fig 1:**
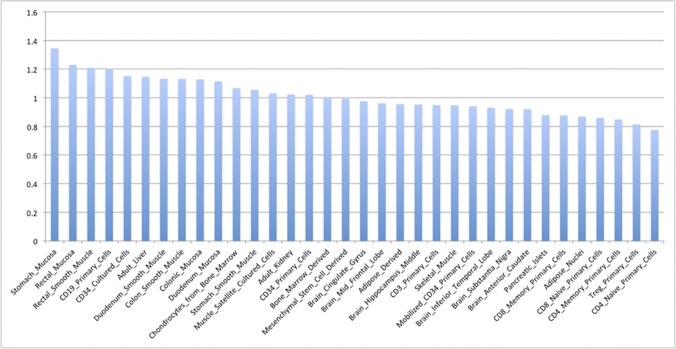
Enrichment of HPV integration sites inside regions marked by H3K4me3 in different cell types. Enrichment for a given cell type is calculated based on overlap between H3K4me3 chip-seq peaks for the cell type and HPV integration sites including 10kb flanks in both directions. Enrichment values are further normalized as described in Methods.

Fig. 1 shows normalized enrichment of HPV integration sites inside regions marked by H3K4me3 in 34 different cell types, sorted from highest to lowest enrichment. Briefly, the normalized enrichment for a given cell type was calculated as a factor of overrepresentation of cell-specific H3K4me3 marks in the proximity of HPV integration sites relative to what would be expected given H3K4me3 occurrence across the genome and across cell types (details provided in Materials and methods). Table 1 shows the relative enrichment values and the p-values. The two most highly enriched cell types (mucosa of the stomach and mucosa of the rectum) are both mucosa cells, and all four mucosal cell types in the dataset were found among the ten most enriched cell types. Cervical mucosal epithelial cells were not a part of the dataset. However, as shown in Table 2, there is a relatively high base pair level similarity between H3K4me3 coverage for the four mucosa cell types for which we have data, suggesting that H3K4me3 in cervical mucosal cells will likely also have a quite high similarity at the base pair level. The consistently high enrichment observed for mucosa cells thus suggests that HPV integration sites are also enriched in regions having H3K4me3 modifications in cervical mucosa epithelial cells.

**Table 1:** Relative enrichment of HPV integration sites within regions marked by H3K4me3 across cell types.

Cell	Relative enrichment	p-value
**Stomach Mucosa**	**1,35**	**0,016**
**Rectal Mucosa**	**1,23**	**0,035**
Rectal Smooth Muscle	1,21	0,032
CD19 Primary Cells	1,20	0,152
CD34 Cultured Cells	1,15	0,192
Adult Liver	1,15	0,257
Duodenum Smooth Muscle	1,13	0,088
Colon Smooth Muscle	1,13	0,084
**Colonic Mucosa**	**1,13**	**0,208**
**Duodenum Mucosa**	**1,11**	**0,152**
Chondrocytes from Bone Marrow Derived Mesenchymal Stem Cell Cultured Cells	1,07	0,208
Stomach Smooth Muscle	1,06	0,287
Muscle Satellite Cultured Cells	1,03	0,356
Adult Kidney	1,02	0,396
CD34 Primary Cells	1,02	0,455
Bone Marrow Derived Mesenchymal Stem Cell Cultured Cells	1,00	0,436
Mesenchymal Stem Cell Derived Adipocyte Cultured Cells	0,99	0,426
Brain Cingulate Gyrus	0,98	0,614
Brain Mid Frontal Lobe	0,96	0,584
Adipose Derived Mesenchymal Stem Cell Cultured Cells	0,96	0,723
Brain Hippocampus Middle	0,95	0,594
CD3 Primary Cells	0,95	0,693
Skeletal Muscle	0,95	0,574
Mobilized CD34 Primary Cells	0,94	0,644
Brain Inferior Temporal Lobe	0,93	0,634
Brain Substantia Nigra	0,92	0,673
Brain Anterior Caudate	0,92	0,762
Pancreatic Islets	0,88	0,752
CD8 Memory Primary Cells	0,88	0,812
Adipose Nuclei	0,87	0,901
CD8 Naive Primary Cells	0,86	0,842
CD4 Memory Primary Cells	0,85	0,743
Treg Primary Cells	0,81	0,743
CD4 Naive Primary Cells	0,78	0,941

All four mucosal cell types in the dataset are among the top ten cells. Enrichment values are normalized so that a value of 1 corresponds to the average across all cell types (details provided in text). P-values are computed conservatively based on the relative enrichment, and are not adjusted for multiple testing.

**Table 2:** Similarity of H3K4me3 segments between different mucosal cell types.

	Unique to each mucosal cell type	Shared with one mucosal cell type	Shared with two mucosal cell types	Shared with all three mucosal cell types
Colonic mucosa	17%	40%	33%	9%
Duodenum mucosa	21%	40%	31%	8%
Rectal mucosa	24%	39%	29%	8%
Stomach mucosa	14%	38%	36%	12%

Approximately one fifth of H3K4me3 segment coverage is unique to one cell type, showing strong similarity between different mucosal cell types.

In order to exclude the possibility of methodological bias (as the sites included in our analysis are identified by methodology detecting active integration sites), a dataset reported in a study by Schwarz and colleagues [12], comprising both active and inactive sites, were used for result confirmation. The outcomes were similar and the conclusion the same as from our primary dataset.

### Enrichment of DNase hypersensitivity sites

Public data were also available for DNase hypersensitivity in various tissues. We used these data to examine whether we could find signs of preferential HPV integration in certain tissues. Table 3 shows the relative enrichment of HPV integration sites within regions marked as DNase hypersensitive across cell types. HPV integration sites were enriched within DHSs of several epithelial cell types, as demonstrated by the fact that all top highly enriched entries represent normal epithelial states, indicating that a common denominator for integration is this cellular organization.

**Table 3:** Relative enrichment of HPV integration sites within regions marked as DNase hypersensitive across cell types.

Cell type	Relative enrichment
NHEK (normal human epidermal keratinocytes*)	1,78684758
SAEC (small airway epithelial cells)	1,7337304
HMEC (human mammary epithelial cells)	1,73121465
PrEC (prostate epithelial cells)	1,66412745
vHMEC (variant of mammary epithelial cells)	1,65127306
HEEpiC (esophageal epithelial cells)	1,63199384
A549 (tumor cells from alveolar basal epithelium)	1,38060164
HCT116 (intestinal epithelial cancer cells)	1,32222737
HeLa (cervical epithelial cancer cells)	1,2858303
PANC1 (pancreatic epithelial cancer cells)	1,19060811
…	
*lowest 10*:	
Th1_2 (T helper cells)	0,84425773
Fetal brain_1	0,84189575
Fetal spinal cord_3	0,83960345
BE2_C (human brain neuroblastoma cells)	0,83553399
Fetal skin_1	0,8314366
Fetal heart_2	0,82877819
Fetal adrenal_2	0,82059539
NHDF_Neo (normal human dermal fibroblasts)	0,82015982
Fetal brain_2	0,80743362
NT2_D1 (human testicular embryonic carcinoma cells)	0,77336166

The table shows the cell types with the ten highest and ten lowest enrichment values. A table of all 147 analyzed cells is provided at the supplementary Galaxy Page. The enrichment values are normalized in the same way as in Table 1 (see text).

* Epidermal keratinocytes are highly specialized epithelial cells.

All results can be inspected in detail and reproduced at the following Galaxy Page: https://hyperbrowser.uio.no/dev2/u/sandve/p/hpv

## Discussion

A certain overrepresentation of HPV integration sites in transcriptionally active regions, fragile sites and chromosomal instability regions has first of all been attributed to the availability of these regions for the integration event. A bias may also reflect greater susceptibility of these regions to integration-induced chromosomal alterations [29,30] or altered gene expression [13,14]. Summarizing previous reports, it remains unclear which factors are the most important, and the dynamics of HPV chromosomal integration is essentially not known. Nevertheless, a general understanding is that the integration event partially is a consequence of a certain level of chromosomal instability caused by the viral E6 and E7 proteins.

Here, we provide statistical data strongly implying the importance of transcriptionally active regions in the process of HPV integration. The integration sites included in our analysis coincide with transcriptionally active regions (shown through H3K4me3 enrichment) in the chromatin of mucosal epithelial cells (Fig. 1 and Table 1), indicating that these regions are more ‘available’ for integration in these cell types. This was further supported by the finding that DHSs were overrepresented for integration in epithelial cells (Table 3). As can be seen, normal epithelial cells appear most enriched, compatible with HPV integration being an early event in the carcinogenesis process. Our data also confirm fragile sites as common targets, with a statistical significant outcome in terms of overrepresentation.

Based on the present reported importance of transcriptionally active regions, we speculate that the common finding of integration sites within or in the vicinity of genes simply is a consequence of the integration event tending to occur in actively transcribing DNA. More importantly, the data included in the analyses (H3K4me3 methylation data) is representing normal cells, not cells under malignant transformation, suggesting that integration is an early event in the carcinogenesis, more than a late product of chromosomal instability. If the viral integrations were more likely to occur in destabilized regions of the DNA as a result of prior genomic instability, a more random distribution of the integration sites would be expected. A matter of debate has indeed been related to when the integration occurs during the stages of pre-cancerous disease. However, conclusions drawn may depend on the method used, and whether the integration process is a late event [31] or also occurs at earlier stages [20,32–35] has been controversial. A common method used for the detection of integrated HPV is the APOT (amplification of oncogene transcripts) assay, and the assumption that integration is a late event is to some extent based on results found by this method. However, when using APOT, only active viral integration sites are investigated, not taking transcriptionally silent sites into consideration [36]. When the viral genome integrates, it becomes a target for cellular control mechanism, as for example host-specific DNA methylation, which in turn results in a down-regulation of the transcriptional activity [37]. Thus, one hypothesis has emerged suggesting that transcriptionally inactive integrants would be expected to remain ‘latent’ until subsequent events abolish transcriptional inhibition [5]. Such silent integrates are demonstrated in a recent paper by Schwarz and colleagues, applying next-generation sequencing both at the level of DNA and the level of mRNA [12].

The sites included in our analysis [7,8] are identified by methodology detecting active integration sites only (APOT). In order to exclude the possibility of methodological bias, as our main finding is that integration sites coincide significantly with DNA that is transcriptionally active, we wanted to assess whether this outcome could be confirmed by analyzing a dataset comprising both active and inactive sites. Such a dataset was found in the study mentioned above by Schwarz and colleagues [12] and analyzed. The outcome was similar and the conclusion the same as from our primary dataset (data shown at the supplementary Galaxy Page).

It has for many years been speculated whether integration may be used as a progression marker for cervical carcinogenesis. Based on our findings, suggesting that integration is an early event more than a late event, it remains to be elucidated which integrations will lead to progression and which will not. Additional events are apparently necessary for cancer progression, and consequently, additional markers are needed in order to predict further diagnosis. For example, it is reasonable to think that a mechanism of selection pressure is involved for integrations in or in the vicinity of cancer-related genes. In other words, if the integration is an early event as suggested here, the detection of integrated virus may, together with other markers, identify underlying severe disease at an earlier stage than what is possible today. Whether there are differences between different HPV types in terms of integration mechanisms remains to be investigated. New technology as next-generation sequencing will undoubtedly speed up the identification and mapping of HPV integration sites, adding important information to the process of HPV-induced carcinogenesis.
